# Exclusive breastfeeding practice and its associated factors among children aged 6–23 months in Woldia Town, Northwest Ethiopia

**DOI:** 10.4314/ahs.v21i4.46

**Published:** 2021-12

**Authors:** Desale Sisay Yimer, Omer Seid Adem, Mastewal Arefayene, Tefera Chanie, Melese Linger Endalifer

**Affiliations:** 1 Public Health Nutritionist, Woldia General Hospital, Woldia, Ethiopia; 2 Department of Nutrition, College of Medicine and Health Science, Bahir Dar University, Bahir Dar, Ethiopia; 3 Department of Public Health, College of Medicine and Health science, Wollo University, Dessie, Ethiopia; 4 Department of Public Health, College of Health Science, Woldia University, Woldia, Ethiopia

**Keywords:** Exclusive Breastfeeding, Breastfeeding practice, Breastfeeding Promotion

## Abstract

**Background:**

Inappropriate practice of exclusive breastfeeding (EBF) is still a major problem worldwide.

**Objective:**

To identify exclusive breastfeeding practice and its associated factors among children aged 6–23 months in Woldia Town

**Methods:**

A Community based cross-sectional study was carried out.Interviewer-administered questionnaire was utilized to collect the data. The questionnaire was adapted from the 2016 Ethiopia Demographic and Health Survey. Hosmer-Lemeshow model was fitted at a P-value of 0.91. Finally, Variables having P-Value <0.05 with 95% CI was considered as significant factors.

**Result:**

The prevalence of exclusive breastfeeding practice was 46.2% (95% CI: 42.0, 49.8). Being employed mothers (AOR=0.62,95% CI:0.44,0.87), being head of a household (AOR=0.52,95% CI:0.32,0.83), not g getting husband support (AOR:0.5,95%CI:0.34,0.74), not fed colostrum's (AOR:0.36,95%CI:0.23,0.57), not affected by traditional belief (AOR:3.59, 95% CI:2.09–6.17) shows significant association with Exclusive breast feeding practice.

**Conclusion:**

The prevalence of exclusive breast feeding practice was relatively lower than the National prevalence. Some demographic variable and traditional beliefs were significantly associated with exclusive breast feeding. Designing model policies that empower the role of women and eradicating bad traditional beliefs in the community is fundamental step.

## Background

Exclusive breastfeeding practices (EBF) is defined as the child takes only breast milk and no additional food, water, or other liquid (except medicine and vitamins if needed) till six months of age^1^, ^2^. Both the World Health Organization (WHO) and the United Nation International Children's Emergency Fund (UNICEF) recommended exclusive breastfeeding for the first six months after birth^3^.

Breastfeeding is considered a first immunization since it protects children from communicable and non-communicable diseases^1^. Human breast milk is naturally balanced nutritious food for infants. Really; it is an ideal food for a child's survival, growth, and development^4^. Feeding A human breast milk in the first six months of life is the most effective evidence-based public health intervention to reduce child morbidity and mortality^5^. It reduces the occurrence of childhood asthma, cancer, obesity, diabetic mellitus, and cardiovascular diseases. Furthermore; it improves cognitive performance and survival rate in their later life as compared to children who fed formula foods^6^, ^7^,^8^. The benefit of Human breast milk is beyond the child health; it stabilizes the household as well as the country economy and at the large it have a great role to build productive citizen6, 9–11. About 1.4 million deaths of under five years children can be prevented annually through implementing optimum exclusive breastfeeding practice^12^. Nonexclusive breastfeeding during the first six months of life impairs children's mental and social development^13^, ^14^. More than 10 million under-five children die each year worldwide, 41% of the deaths occur in Sub-Saharan Africa, and 34% in South Asia. Inappropriate breastfeeding practice combined with high levels of disease is a major cause for childhood death^15^, ^16^.

In the past different scholars assess the magnitude and determinant factors of exclusive breastfeeding in different parts of the world^17^–^20^. Intensively socio-demographic factor such as the older the mother, being married, being well educated, and being in a higher income class^17^, ^18^, ^21^–^23^, child age^19^, ^22^, ^24^, sex of the infant^23^ and family size^25^, ^26^ were identified as contributing factors for the duration of breastfeeding.

Information on the benefits of exclusive breastfeeding^20^,^24^, ^27^, place of delivery^21^, ^28^, Infant feeding counseling^18^, ^22^, antenatal care visit^17^, ^29^, employment status of mother^27^,^30^,^17^, ^18^, ^31^ and short maternity leave^17^, ^32^ were reported as determinant factor exclusive breast feeding practice.

The rate of exclusive breastfeeding in Ethiopia was 58% according to the 2016 Ethiopia Demographic and Health Survey (EDHS)^33^. Currently, 24% of infant death is due to poor breastfeeding practices in Ethiopia^28^,^34^.

In fact many scholars depicted that there is a variation on the magnitude as well as determinant factors across the geographic region, economic class, and society arrangements^35^–^40^. Additionally we plan to add new variables such as male involvement. That is why we are intended to assess exclusive breastfeeding practice and its associated factors among children aged 6–23 months in Woldia town; Northeast, Ethiopia.

## Methods and Materials

### Study Area and Period

A community-based cross-sectional study was conducted from November 04/2017 – January 04/2018. Woldia is the epicenter of North Wollo Zone and located in the Amhara region. It is far from 370 km East of Bahr Dar and 520 km North of Addis Ababa. The town has a total population of 75446 of which 38167 are males and 37279 are females. There about 2454 children aged 6–23 months in Woldia town exist during the study period41. Regarding the health institution profile in the town one public hospital, two public health centers, and so several private pharmacies and clinics gives healthcare services to the community.

### Source population

The source population for this study was all mothers with children aged 6–23 months in Woldia town.

### Study population

The study participants were those mothers with children aged 6–23 months in Woldia town in the selected four kebeles.

### Eligibility criteria

#### Inclusion criteria

Mother who lived at least six months in Woldia town.

#### Exclusion criteria

Those mothers who cannot communicate verbally and mothers who were unable to breastfeed due to medical diagnosed breast problems.

### Sample size determination

The sample size was determined by using single population proportion formula. Here under was an assumption considered for sample size calculation. Prevalence of EBF =0.65(19);Z α/2= 1.96, w= margin of error (5%) =0.05 and a design effect of 2. The final sample size gives a total of 700 study participants.

### Sampling Technique

A multistage simple random sampling technique was applied. From ten kebeles (the smallest administrative unit in Ethiopia) of Woldia town, fours were selected randomly. The list of mothers with children aged 06–23 months was collected from kebeles health extension workers. Then the data was entered into the computer and participants were selected through the OPEN EPI random generating system. Proportional allocation of study participants were made to each four kebeles.

### Study variables

The dependent variable was exclusive breastfeeding practice. The practice of EBF was assessed by asking the mother “have you exclusively breastfed your child during the first 6 months of life?” The response had binary outcome (Yes/No). The dependent variable was gathered through recall method by asking retrospectively and the method was recommended by WHO. The independent variables were sociodemographic obstetric and child health characteristics.

### Operational definitions and definition of terms

#### Separation time

A time duration in a day which a mother and a child separated due to work conditions in the first 6 months of child age.

#### Traditional beliefs towards exclusive breastfeeding

This means when the study participants were influenced by cultural beliefs regarding exclusive breastfeeding. If the mother is practiced non-exclusive breastfeeding due to the effect of traditional belief it was categorized as the mother was affected by tradition.

#### Wealth index

The wealth index was used to assess the household economic status and categorized as poor, middle and rich.

#### Husband support

When a husband allowed his wife to practice EBF.

#### Antenatal care visit

If pregnant woman received at least one antenatal care visit, they considered as “had antenatal care visit” and pregnant women who did not attend at all recorded as “no antenatal care visit”.

#### Postnatal care visit

A pregnant woman received at least one postnatal care visit, they considered as “had postnatal care visit” and pregnant woman did not attend at all recorded as “no postnatal care visit”.

#### Immunization status

A child is considered as immunized when they vaccinated at least one vaccine in the past 24 months unless otherwise, they were considered as non-immunized.

### Data collection methods and techniques

Interviewer-administered structured questionnaire was utilized. The questionnaires had three components which consists of socio-demographic variables, child health-related characteristics, and mother's health-related characteristics. The questionnaire was prepared in English, then translated to Amharic and back-translated into English with a professional language translator. The questionnaire was adapted from the 2016 Ethiopia Demographic and Health Survey report. The training was given for one day to the data collectors by the principal investigator. Five percent of the questionnaire was pre-tested in the Kobo district and an amendment was done before the actual data collection. The data collector goes to the home of the participants and by using a code given during the sampling period, they commenced data collection. The interview was held separately to maintain the confidentiality of the respondents. In each household, only one woman with a child 06–23 months of age was interviewed. Furthermore, if the mother had two eligible children age less than two years and the younger one was considered. Each questionnaire was checked for its completeness and consistency at the end of the day.

### Data analysis

The data was entered in EpiData version 3.1 and analyzed in SPSS version 21 software. The wealth index was analyzed through principal component analysis (PCA). A descriptive analysis was carried out to present the data. Variables having P-value < 0.2 in the binary logistic regression were further analyzed through a multivariable logistic regression model to control potential confounders. Hosmer-Lemeshow was checked to examine model fitness. Finally, variables having a p-value of <0.05 with 95% CI counted as significant predictors for the exclusive breastfeeding practices.

## Results

### Socio-demographic characteristics

Among 700 mothers, 634 were involved in this study with a response rate of 90.5%. From the total mothers, 514 (84.5%) got married, 291(45.9%) have secondary and above education level and 330(52.1%) were lived in a family size of 2–4 peoples (Table 1).

**Table 1:** Socio - demographic characteristics of study participants in Woldia town, Northeast Ethiopia, 2017

Variables	Exclusive breastfeeding practice		Total (N=634)
	Yes (N=293)	No(N=341)	
**Age of mothers (in years)**			
<20	10(1.6%)	14(2.2%)	24(3.8%)
20–34	208(32.8%)	225(35.5%)	433(68.3%)
35–49	75(11.8%)	102(16.1%)	177(27.9%)
**Marital status of** **the Mother**			
Married	251(39.6%)	263(41.5%)	514(84.5%)
Single	20(3.2%)	25(3.9%)	45(7.1%)
Divorced/Widowed	22(3.4)	53(8.4%)	75(11.8%)
**The education level of the** **mother**			
Illiterate	11(1.8%)	10(1.6%)	21(3.3%)
Primary education	77(12.1%)	77(12.1%)	154(24.3%)
Secondary education	77(12.1%)	91(14.4%)	168(26.5%)
More than secondary	128(20.2%)	163(25.7%)	291(45.9%)
**Occupation of mothers**			
Employed	134(21.1%)	183(28.9%)	317(50%)
**Head of household**			
Mother	35(5.5%^	85(13.4%)	120(18.9%)
Father	258(40.7%)	256(40.4%)	514(81.1%)
**Child sex**			
Male	171(27%)	183(28.9%)	354(55.8%)
Female	122(19.2%)	158(24.9%)	280(44.2%)
Child age(in months)			
6–11	127(20%)	161(25.4%)	288(45.4%)
12–17	104(16.4%)	119(18.8%)	223(35.2%)
18–23	62(9.8%)	61(9.6%)	123(19.4%)
**Family size(in number)**			
5	149(23.5%)	181(28.5%)	330(52.1%)
>5	144(22.7%)	160(25.3%)	304(47.9%)
**Wealth quintile**			
Poor	108(17%)	145(22.9.%)	253(39.9%)
Middle	63(9.9%)	68(10.7%)	131(20.7%)
Rich	122(19.3%)	128(20.2%)	250(39.5%)

### Maternal and child health related characteristics

A total of 588(92.7%) participant attend antenatal care (ANC), 573(90.4%) were delivered in health facility, 625(98.6%) children were immunized and 570(89.9%) attend postnatal care visit (PNC) follow up (Table 2).

**Table 2:** Maternal child health services utilization characteristics of the study subjects in Woldia town, Northeast Ethiopia, 2017

Variables	Exclusive breastfeeding practice		Total (N=634)
	Yes(N=293)	No(N=341)	
**Place of delivery**			
Home	18(2.8%)	43(6.8%)	61(9.6%)
Health facility	275(43.4%)	298(47%)	573(90.4%)
**Mode of delivery**			
Spontaneous vaginal delivery(SVD)	192(30.3%	225(35.5%)	417(65.8%)
Caesarean section (CS)	101(15.9%)	116(18.3%)	217(34.2%)
**Birth interval**			
1 year	17(2.7%)	24(3.8%)	41(6.5%)
2–3 year	125(19.7%)	135(21.3%)	260(41%)
4–5 year	129(20.3%)	160(25.2%)	289(45.6%)
More than 6 year	22(3.5%)	22(3.5%)	44(6.9%)
ANC follow up			
No	19(3%)	27(4.3%)	46(7.3%)
Yes	274(43.2%)	314(49.5%)	588(92.7%)
PNC follow up			
No	22(3.5%)	42(6.6%)	64(10.1%)
Yes	271(42.7%)	299(47.2%)	570(89.9%)
Was the child immunized?			
No	1(0.2%)	8(1.3%)	9(1.4%)
Yes	292(46%)	333(52.5%)	625(98.6%)
Was the mother got an advice on EBF?			
No	29(4.6%)	50(7.9%)	79(12.5%)
Yes	264(41.6%)	291(45.9%)	555(87.5%)
Husband support			
No	67(10.6%)	67(10.6%)	203(32%)
Yes	226(35.6%)	205(32.3%)	431(68%)
Feed colostrum's			
No	31(4.9%)	92(14.5%)	123(19.4%)
Yes	262(41.3%)	249(39.3%)	511(80.6%)

### Prevalence of exclusive breast feeding practice

The prevalence of exclusive breastfeeding practice was 46.2% (95% CI: 42.0, 49.8). From the total study participants; 470 (74.1%) of children fed breast milk eight and more times in 24 hours. The reasons mentioned as a barrier topractice exclusive breastfeeding were inadequate breast milk production, traditional beliefs, work conditions, short maternity leave, and lack of child feeding facility at the workplace (Table 3).

**Table 3:** Exclusive breastfeeding practice among children aged 6–23 months in Woldia Town, Northeast Ethiopia, 2017

Variables	Frequency (%) Tot(N=293) No(N=341)
**Have you exclusively breastfed your child during the first 6** **months?**	
Yes	293 (46.2%)
No	341 (53.8)
**Is there any traditions that hinder EBF practice?**	
No	544(85.8%)
Yes	90(14.2%)
**Did you believe breast milk is sufficient for the** **Infants?**	
No	242(38.2%)
Yes	392(61.8%)
**Frequency of Breastfeeding in 24 hour**	
Less than eight times	164(25.9%)
Eight and more tha	470(74.1%0
**Reasons for not practicing exclusive** **breastfeeding**	
**Work condition**	
No	462(72.9%)
Yes	172(27.1%)
**No adequate breast milk production**	
No	426(62.5%)
Yes	208(32.8%)
**Short maternity leave**	
No	556(87.7%)
Yes	78(12.3%)
**Absence of child feeding facility at workplace**	
No	497(78.4%)
Yes	137(21.6%)
**Non-flexibility of work time**	
No	579(91.3%)
Yes	
Yes	55(8.7%)

### Factors associated with exclusive breastfeeding practice

Employment status of the mothers, being head of household, getting husband support, feeding colostrum and traditional beliefs towards breastfeeding were predictors of EBF practice.

Employed mothers were 38 % times less likely to practice exclusive breastfeeding as compared to non-employed mothers (AOR=0.62, 95 % CI: 0.44, 0.87). A mother who did not got husband support decreases the occurrence of EBF by 50% as compared to the counterparts (AOR=0.50,95 %CI:0.34,0.74). Those mothers who fed colostrum were 64 % less likely to practice EBF as compared to mothers who fed colostrum previously (AOR=0.36,95%CI:0.23,0.57). Those mothers who didn't affected by the traditional beliefs were practice EBF more than three folds relative to mothers affected by traditional beliefs (AOR=3.59,95%CI:2.09,6.17). The likelihood of EBF practice among mother headed family was decreased by 48% as compared to the father headed family (AOR=0.52,95%CI:0.32,0.83) (Table 4).

**Table 4:** Bi-variable and multivariable logistic regression analysis of exclusive breastfeeding practice among children aged 6–23 months inWoldia Town, 2017

Variables	EBF practice		COR(95 % CI)	AOR(95%CI)
	Yes	No		
**Employment status**				
Employed	134	183	0.73(1.01,1.88)	0.62(0.44,0.87)**
Non-employed	159	158	1	1
**Marital status**				
Married	251	263	1	1
Single	20(3.2%)	25	0.84(0.45,1.55)	1.11(0.45,2.75)
Widowed/divorced	22	53	0.44(0.26,0.74)	1.16(0.44,3.04)
**Head of household**				
Mother	35	85	0.41(0.27,0.63)	0.52(0.32,0.83)**
Father	258	256	1	1
**Educational level of mothers**				
Illiterate	11	10	1.40(0.58,3.40)	1.67(0.58,4.86)
Primary education	77	77	1.27(0.86,1.88)	1.42(0.84,2.39)
Secondary education	77	91	1.08(0.74,1.58)	0.98(0.61,1.59)
More than secondary	128	163	1	1
**Place of delivery**				
Home	18	43	0.45(0.26,0.81)	0.73(0.38,1.40)
Health facility	275	298	1	1
**Sex of child**				
Male	171	183	1.21(0.88,1.66)	1.24(0.89,1.74)
Female	122	158		1
**Husband support**				
No	67	136	0.45(0.32,0.63)	0.50(0.34,0.74)**
Yes	226	205	1	1
**Partner get advice**				
No	61	121	0.48(0.33,0.68)	0.88(0.53,1.44)
Yes	232	220	1	1
**Fed colostrum's**				
No	31	92	0.32(0.21,0.50)	0.36(0.23,0.57)**
Yes	262	249	1	1
**Affected by traditional beliefs**				
No	272	272	3.29(1.96,5.51)	3.59(2.09,6.17)**
Yes	21	69	1	1

## Discussion

In the current study, the prevalence and determinant factors of EBF practice was assessed in Woldia town. This finding reports the life time prevalence exclusive breast feeding among under two years children. The prevalence of exclusive breastfeeding practice was 46.2% (95% CI: 42.0,49.8). Employment status of the mother, being head of household, getting husband support, fed colostrum's and traditional beliefs towards breast milk feeding showed a significant association with EBF. The overall prevalence of exclusive breastfeeding practice was 46.2 %. This result was lower than EDHS 2016 33, Debre Tabor town^42^, Endereta Wereda^28^, Goba district^17^, Goffa district-southern Ethiopia(78%)^43^, Debre Markos (60.8%)^44^, Hosanna Town(74%)(45), Dilla town(26) and Ghana^46^. The difference might be; the previous researchers collect the data among children aged 0–6 months, quite sure this increases the prevalence and the current study is prone to recall bias which may lead the result over or underestimation.

In the current finding the prevalence of EBF practice was comparable with studies conducted in different parts of Ethiopia^25^, ^31^, ^47^, ^48^. On the other hand, the prevalence of exclusive breastfeeding practice in the current study was higher than studies conducted elsewhere^29^, ^30^,^32^, ^49^. This gap may result from study design differences, socioeconomic variation, and the variation in the study participants included in the study sample.

Getting husband support was significantly associated with EBF. The finding was in line with the study done in Nigeria^49^. Predominantly husbands have a great role to increases their degree of freedom in social, economic, and cultural aspects; so those mothers who got positive response from her husband increases the to practice EBF freely.

Mothers who did not affected by traditional beliefs were three times more likely to practice exclusive breastfeeding. This finding was in agreement with studies done in Australia^50^, Zambia^24^ which states that socio-cultural determinants influences breastfeeding patterns. Traditionally introducing liquids or food before six months is recommended and considered as good habit in the majority of Ethiopian community. As a result, those mothers who overcome these beliefs practiced EBF effectively and efficiently.

Households headed by mothers affect the practice of exclusive breastfeeding negatively. The finding was similarly reported study conducted in Mecha District^47^ and Hosanna town of Ethiopia^45^. This might be the effect of the additional workload of the mother which decreases their time to feed the child. In such family mothers could be burdened more than tripled role that means leading a family, social role, economic role and miscellaneous role.

Mothers who did not give colostrum's during the first hour after birth were less likely to practice feeding breast milk exclusively than who gave colostrum timely. This finding was similarly reported studies conducted in different parts of Ethiopia^22^, ^29^, ^31^, ^47^, ^51^. This might be early suckling of colostrum initiate the production of milk that pushes the mother to feed their child breast milk. Employed mothers were less likely to practice EBF than unemployed mothers. This finding was in line with studies done in Injibara, Ethiopia^19^, and Malaysia^48^. This might be an indication for absence of onsite child care facilities, non-flexibility of job time, not initiating mother's friendly policies at the workplace that support breastfeeding. Additionally, those employed mothers might have a good income which gave a chance to bought formula milk. Even though the current study had a number of strengths; it have some drawbacks. For instance, the study is not free from recall and social desirability bias. Additionally the cross-sectional nature of the study makes it difficult to assess disease causation.

## Conclusion

The prevalence of exclusive breastfeeding practice was relatively lower than the national EBF figure. Being employed, getting husband's support, being head of household, feeding colostrum's, and traditional belief towards breast milk feeding in the community were significantly associated with exclusive breastfeeding practice. The practice of non-exclusive breastfeeding practice is too large; it needs the eyes of the government. A holistic approach that improves the well-being, lifestyle and educational status of the mother is mandatory. Specifically designing model policies that empower the role of women in the community and reducing the work burden of women would be the first option. Lastly; the government should give emphasis on eradicating bad traditional beliefs towards exclusive breast feeding in the community.

## Abbreviations and acronyms

AOR Adjusted Odds Ratio

ANC Ante Natal care

CI Confidence Interval

COR Crude Odds Ratio

EBF Exclusive Breastfeeding

EDHS Ethiopian Demographic and Health Survey

PNC Post Natal Care

REC Research Ethical Committee

SPSS Statistical Package for Social Science

UNICEF United Nation International Children's Emergency Fund

WHO World Health Organization

## Limitations

The current study is suffered from recall and social desirability bias which may under or overestimate the findings.

## Availability of data and supporting materials

The data can be accessed from the corresponding author based on reasonable request.
